# The (STEM)^2^ Network: a multi-institution, multidisciplinary approach to transforming undergraduate STEM education

**DOI:** 10.1186/s40594-020-00262-z

**Published:** 2021-01-29

**Authors:** Jessica Santangelo, Lawrence Hobbie, Jacqueline Lee, Michael Pullin, Eugenia Villa-Cuesta, Alison Hyslop

**Affiliations:** 1 Hofstra University, 1000 Hempstead Turnpike, Hempstead, NY 11549 USA; 2 Adelphi University, 1 South Avenue, Garden City, NY 11530 USA; 3 Nassau Community College, 1 Education Drive, Garden City, NY 11530 USA; 4 Queensborough Community College, 222-05 56th Avenue, Queens, NY 11364 USA; 5 St. John’s University, 8000 Utopia Parkway, Queens, NY 11439 USA

**Keywords:** Network, Interdisciplinary, Inter-institutional, Collaborations, Transfer students, STEM education transformation

## Abstract

**Background:**

Transforming the culture of STEM higher education to be more inclusive and help more students reach STEM careers is challenging. Herein, we describe a new model for STEM higher education transformation, the Sustainable, Transformative Engagement across a Multi-Institution/Multidisciplinary STEM, (STEM)^2^, “STEM-squared”, Network. The Network embraces a pathways model, as opposed to a pipeline model, to STEM career entry. It is founded upon three strong theoretical frameworks: Communities of Transformation, systems design for organizational change, and emergent outcomes for the diffusion of innovations in STEM education. Currently composed of five institutions—three private 4-year universities and two public community colleges—the Network capitalizes on the close geographic proximity and shared student demographics to effect change across the classroom, disciplinary, institutional, and inter-institutional levels.

**Results:**

The (STEM)^2^ Network has increased the extent to which participants feel empowered to be change agents for STEM higher education reform and has increased collaboration across disciplines and institutions. Participants were motivated to join the Network to improve STEM education, to improve the transfer student experience, to collaborate with colleagues across disciplines and institutions, and because they respected the leadership team. Participants continue to engage in the Network because of the collaborations created, opportunities for professional growth, opportunities to improve STEM education, and a sense that the Network is functioning as intended.

**Conclusion:**

The goal to increase the number and diversity of people entering STEM careers is predicated on transforming the STEM higher education system to embrace a pathways model to a STEM career. The (STEM)^2^ Network is achieving this by empowering faculty to transform the system from the inside. While the systemic transformation of STEM higher education is challenging, the (STEM)^2^ Network directly addresses those challenges by bridging disciplinary and institutional silos and leveraging the reward structure of the current system to support faculty as they work to transform this very system.

**Supplementary Information:**

The online version contains supplementary material available at 10.1186/s40594-020-00262-z.

## Introduction

Despite many calls for the transformation of STEM higher education since at least 1924, changes have been slow and the impacts limited (American Association for the Advancement of Science, 2011; Asai & Bauerle, 2016; Association of American Medical Colleges - Howard Hughes Medical Institute, 2009; Brinton, 1924; National Research Council, 2003; Olson & Riordan, 2012; Seymour & Fry, 2016; Steen, 2005). The Sustainable, Transformative Engagement across a Multi-Institution/Multidisciplinary STEM, (STEM)^2^, “STEM-squared”, Network provides a new model for the transformation of STEM higher education with a goal to make it easier for students to stay in or enter STEM career pathways. The Network addresses the multiple levels at which transformation must occur through the development of collaborations among biology, chemistry, and math faculty at 2- and 4-year institutions. It creates new directions in pedagogical and scholarly collaboration (Boyer, Moser, Ream, & Braxton, 2015) by leveraging inter- and intra-institutional multidisciplinary bridges.

We begin by describing a pathways model to STEM careers (Cannady, Greenwald, & Harris, 2014) and the need for STEM higher education transformation. Then, we introduce three complementary theoretical frameworks underpinning the Network: Communities of Transformation (Kezar & Gehrke, 2015), systems design for organizational change (Watson & Watson, 2013), and emergent outcomes (Henderson, Finkelstein, & Beach, 2010). We then detail the Network’s activities and the integration of the theoretical frameworks into those activities. We discuss the challenges to the systemic transformation that the Network addresses and conclude with evidence of the impacts of the Network on faculty participants. As part of this work, we ask several research questions related to if and how well the Network empowers participants to become change agents and encourages members to collaborate across disciplines and institutions. We further explore the motivations underlying participant decisions to join and continue working within the Network and the Network’s impact on their professional development.

### A pathways model to STEM careers

The traditional leaky pipeline model evokes an image of a single path to a STEM career that narrows as students leave STEM at juncture points, such as high school graduation, declaring a STEM major, or graduating from college. A more complex, but realistic model is a pathways model with multiple entryways, exit points, and re-entry ways into a STEM career (Cannady et al., 2014). This shift from the traditional perspective of a pipeline to a perspective of pathways highlights opportunities to make STEM degrees and careers more accessible and inclusive (Cannady et al., 2014; Tajmel, 2019).

In the pipeline model, the two primary predictors for identifying future STEM professionals are an early interest in pursuing a STEM career and taking calculus in high school. However, only 23% of STEM professionals had both these indicators, with 61% having only one and 16% having neither (Cannady et al., 2014). Even for those students who declare an interest in earning a STEM degree and therefore seem to be following a pipeline model, fewer than 40% ultimately earn a STEM degree (Olson & Riordan, 2012). In addition, of those earning a STEM degree, a disparity exists between students who are White or Asian, 46% of whom complete a STEM degree in 5 years, and minoritized students, of whom only 26.8% do so (Chen, 2013; Huang, Taddese, & Walter, 2000).

Transforming STEM higher education is therefore important both economically, to fill our need for a large, diverse set of STEM professionals (Bureau of Labor Statistics, U.S. Department of Labor, 2012; Carnevale, Smith, & Melton, 2011; Chen, 2013; Cole & Barber, 2009; Olson & Riordan, 2012), and to improve the equity and inclusiveness of our society by creating equitable learning and job attainment opportunities (Chen, 2013; Seymour et al., 2020). The pathways model, in recognizing the multiple trajectories towards STEM careers, can simultaneously provide the environment to help retain students currently interested in STEM careers while encouraging the exploration of STEM for all who might be interested.

**Fig. 1:**
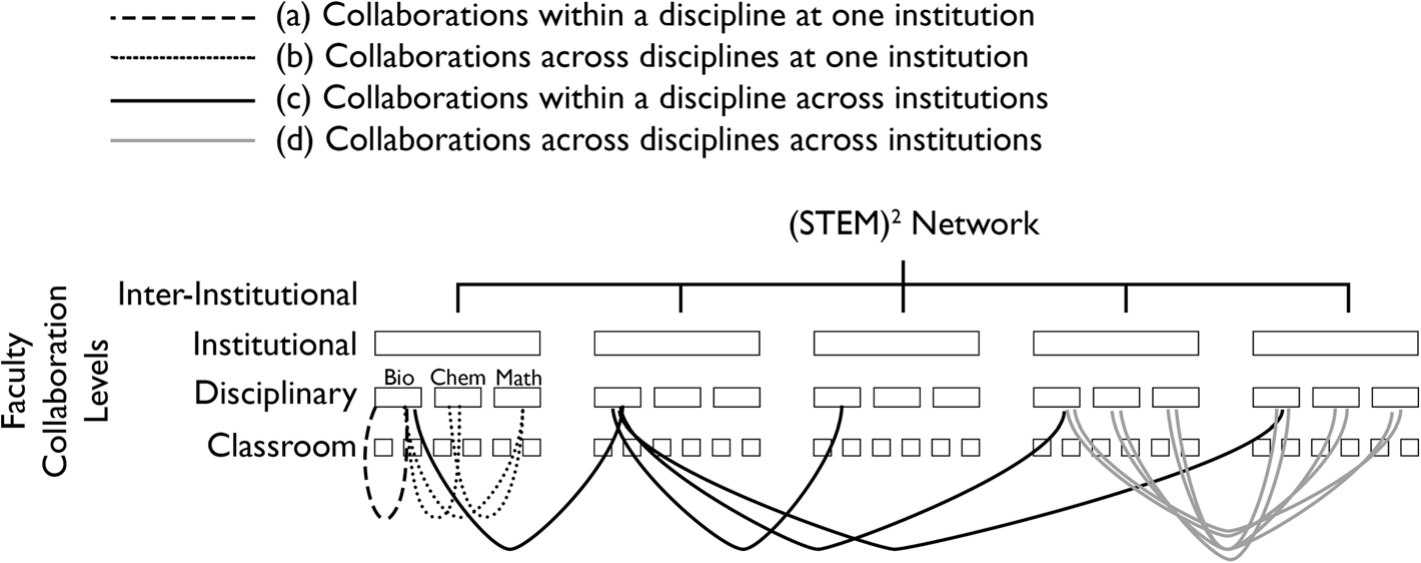
Four levels of faculty collaboration promoted by participation in the (STEM)^2^ Network. The Network spans the classroom, disciplinary, institutional, and inter-institutional levels. It creates on-going collaborations among faculty **a** within a discipline at one institution, **b** across disciplines at one institution, **c** within a discipline across institutions, and **d** across disciplines across institutions. Only a few examples of each collaboration type are illustrated for clarity

The challenge is to restructure institutions to provide an inclusive environment that acknowledges and leverages the multiple pathways to a STEM career. As the ones who directly interact with students, faculty must be stakeholders in the process to initiate and enact these transformations. To impact the culture of STEM higher education, work must be coordinated across the classroom, disciplinary, institutional, and inter-institutional levels (Fig. 1) and be guided by strong theoretical frameworks.

### Theoretical frameworks

The (STEM)^2^ Network’s theoretical foundation unites three frameworks: Communities of Transformation (CoT), systems design for organizational change, and emergent outcomes for the diffusion of STEM innovations.

#### Communities of Transformation

**Fig. 2:**
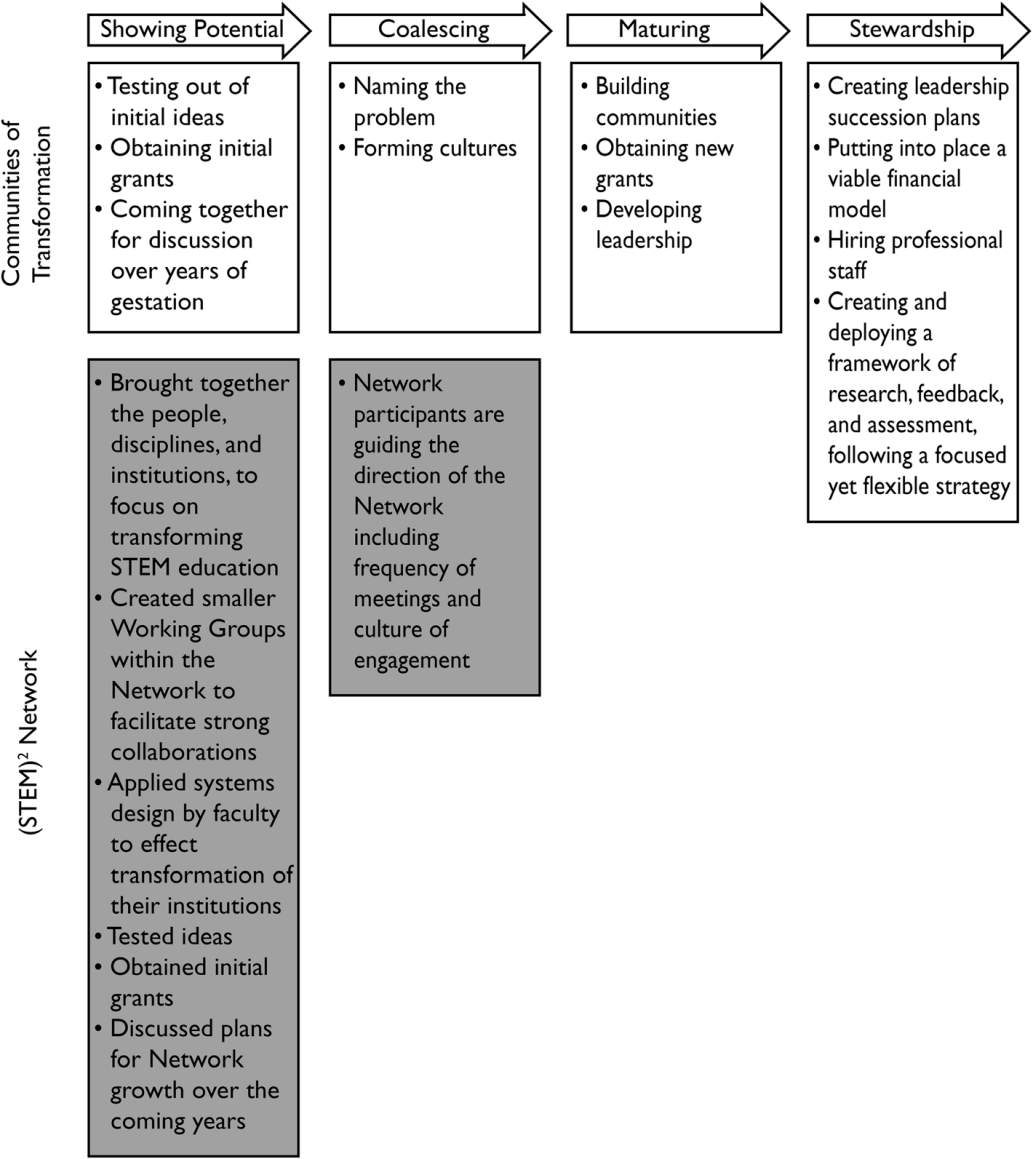
(STEM)^2^ Network Development as a Community of Transformation. The trajectory of development followed by the four existing Communities of Transformation (adapted from Kezar & Gehrke, 2015) aligned with development, to date, of the (STEM)^2^ Network

CoTs are a variant of Communities of Practice that “[explore] philosophically, in deep and fundamental ways, how science is taught” (Kezar & Gehrke, 2015). They simultaneously address transformations involving individual faculty and the broader system. Four CoTs have been identified (Project Kaleidoscope (PKAL, http://www.aacu.org/pkal); Science Education for New Civic Engagements and Responsibilities (SENCER, sencer.net); BioQuest (bioquest.org); Process-Oriented Guided Inquiry Learning (POGIL Project, https://pogil.org/)), all following similar trajectories as they evolved (Fig. 2), suggesting that new CoTs, like the (STEM)^2^ Network, can follow similar steps from their creation to the realization of their goals. Currently, in the “showing potential” phase, the (STEM)^2^ Network is testing ideas, obtaining initial grants, and discussing our plans for Network growth over the coming years.

#### Systems design

Systems design comprises both systems theory and design theory (Watson, Reigeluth, & Watson, 2008; Watson & Watson, 2013). Systems theory views organizations as a system of multiple interacting and interdependent subsystems. Problems and solutions are viewed within the context of the whole system, taking into account the relationships among the subsystems. Given the complexity of higher education institutions, a systems theory approach to institutional change is likely to be more productive than an isolated, piecemeal approach.

Design theory involves the creation of a new system through a process that is holistic, iterative, and involves collaboration among stakeholders (Watson et al., 2008). The primary stakeholders in the (STEM)^2^ Network are faculty. This theoretical framework emphasizes that the Network’s goal is more than the creation of activities, modules, or classes. Rather, the goal is to catalyze transformation within the context of the whole system, creating change that is sustainable, resisting return to the status quo.

#### Emergent outcomes

**Fig. 3:**
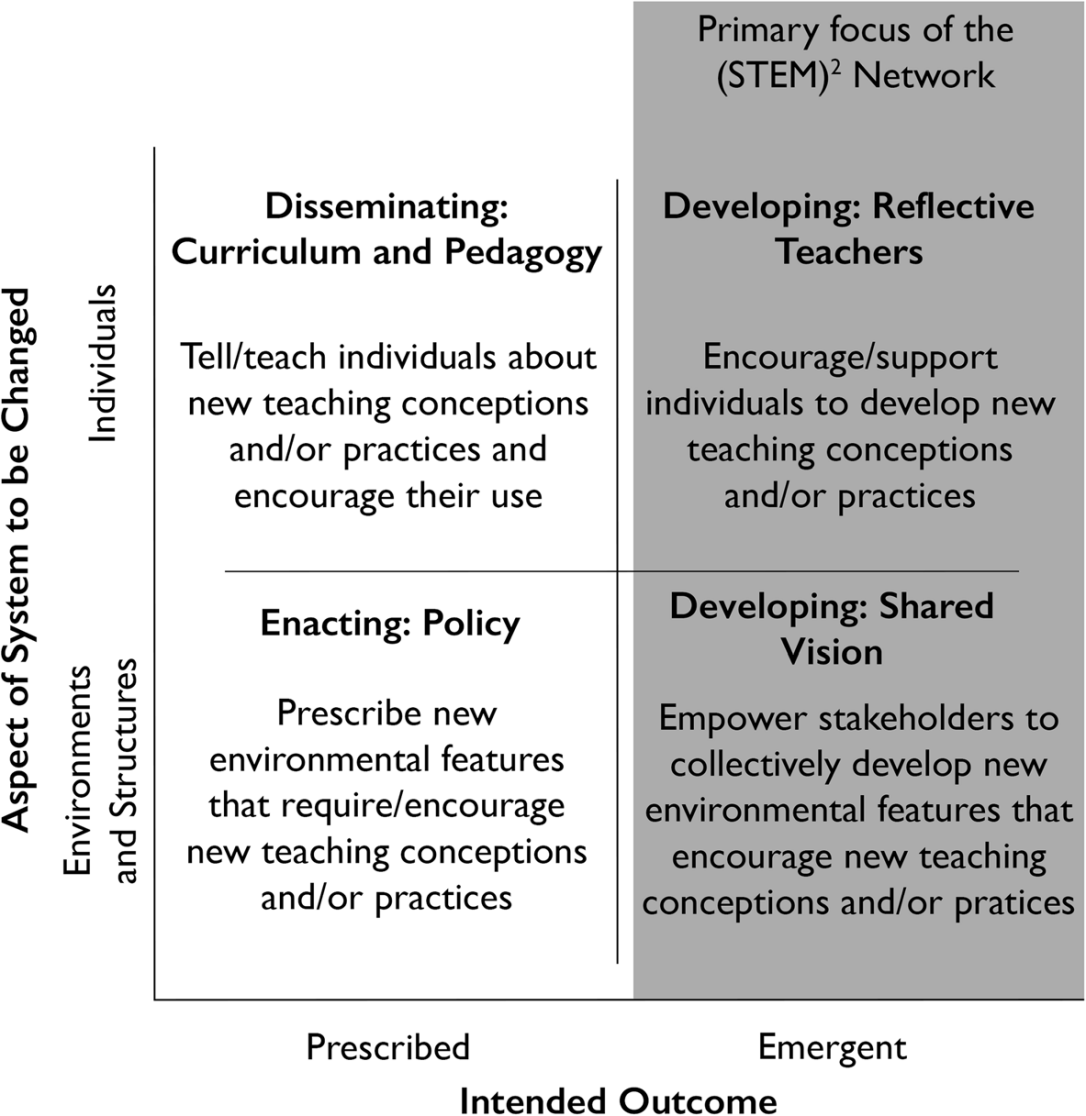
(STEM)^2^ Network and the four change strategies described in Henderson et al. (2010). The (STEM)^2^ Network primarily focuses on emergent outcomes that impact the individuals and the environments and structures in which they teach. Figure modified from Henderson et al. (2010)

Henderson et al. (2010) describe a model of change strategies falling along two axes: the aspect of the system to be changed (Individuals vs. Environments and Structures) and the intended outcome (Prescribed vs. Emergent) (Fig. 3). Most change efforts focus on prescribed outcomes defined by the change agent as desirable prior to initiating the change. These efforts rarely support widespread changes in STEM education, likely because they do not engage the individual in the change process nor do they address the environment and structures in which individuals operate. The (STEM)^2^ Network addresses the lack of diffusion of innovations in STEM education by utilizing an emergent outcomes model. In this model, ideas are generated and implemented by the diverse disciplines and institutions represented in the Network.

### (STEM)^2^ Network description and activities

The (STEM)^2^ Network currently includes two public community colleges, Nassau (NCC) and Queensborough (QCC) Community Colleges, and three private 4-year institutions, Adelphi, Hofstra, and St. John’s Universities, all located on western Long Island, New York, and serving similar student populations, mostly from the New York City metropolitan area. The Network is composed of 25 biology, chemistry, and math faculty from across the five institutions. Many students transfer from NCC and QCC to nearby 4-year institutions. Despite geographic proximity and the existence of articulation agreements, the dialogue between the institutions regarding curricular pathways and courses was previously limited. This limitation placed transfer students at a disadvantage relative to their non-transfer counterparts. The (STEM)^2^ Network, by intentionally including faculty from both 2- and 4-year institutions, promotes course and curricular collaboration to address the needs of these transfer students. In addition, this geographic proximity promotes group identity, cooperation, participation, and decision making (Kiesler & Cummings, 2002), all characteristics important to the development of a synergistic, sustainable network. The Network is leveraging this geographic proximity to formalize previously casual relationships and create new collaborations among faculty to achieve common goals in STEM education.

The (STEM)^2^ Network grew out of several existing formal and informal inter-institutional relationships. A National Science Foundation (NSF) Research Coordination Network - Undergraduate Biology Education (RCN-UBE) grant funded a pilot year that began in January 2020. The Network’s overall mission is to enhance undergraduate STEM education via three related goals:

- Promoting collaboration between geographically proximal community colleges and 4-year institutions;
- Empowering faculty to create change beyond their individual classrooms;
- Creating enduring pedagogical collaborations across STEM disciplines encountered by STEM majors.

To achieve these goals, we brought together the people, disciplines, and institutions to focus on transforming STEM higher education. The outcome we hope to achieve is the creation of multiple, inclusive pathways for students to attain STEM degrees leading to a large, diverse STEM workforce. We created Working Groups within the Network to facilitate strong collaborations and incorporated systems design to empower faculty to transform their institutions.

#### Full (STEM)^2^ Network meetings

The Network meets as an entire group two to three times a year, with multiple smaller subgroup meetings interspersed between the full Network meetings. The full Network meetings are structured as “studio workshops” to promote dialogue, collaboration, and creation (Romice & Uzzell, 2005; Vyas, van der Veer, & Nijholt, 2013). Interdisciplinary and inter-institutional groups are given time to work, share their work, and gather data and feedback from the full Network. Full Network meetings are also used to discuss the theory of and put into practice systems design for organizational change. This area—new for most Network participants—serves as the foundation for communicating and discussing our respective institutions and equips participants to create transformation beyond their classroom.

#### Integration of frameworks

The Community of Transformation framework is ideal for our purposes given the accrual of benefits both to individual CoT members and their institutions. The Network activities are intentionally designed to provide all participants opportunities to co-author publications and grant proposals to further their individual academic careers while simultaneously promoting transformation at their institution. The collaborations built through the overall Network and the Working Groups facilitate on-going interactions. In this way, the CoT framework concurrently supports individual faculty growth and institutional transformation.

The (STEM)^2^ faculty utilize systems design and design theory in a collaborative, iterative process to create lasting paradigm shifts. The systems design work began with systems mapping led by consultants in the field. Participants worked in institutional teams to build visual diagrams of STEM pathways at each institution using rich pictures (Supplementary Material). Rich pictures are created on large sheets of paper, providing the space for a group to discuss, share, and engage in reflection on the context on which they are focusing (Bell & Morse, 2013). For Network participants, this focus was pathways to STEM degrees at their institutions. The pictures included the people, units, relationships, processes, and barriers that arise along the pathways to STEM degree attainment. They provided participants with a visual depiction of the component parts and their interactions, making it easier to identify barriers and potential leverage points for change.

Rich pictures were followed by teams creating influence diagrams (Supplementary Material), visuals that help identify connections and leverage points, and make decisions within a complex system (Diffenbach, 1982). These rich pictures and influence diagrams provided a starting point to develop an action plan. For example, one institutional team realized that including statistical computing and graphics in introductory courses across the curriculum would help students integrate their learning, and another institutional team is planning to use their rich picture to present issues to their new Provost and President.

The rich pictures and influence diagrams provided a starting point from which to apply systems change and theory of change frameworks (Kania, Kramer, & Senge, 2018; Rogers, 2014). The systems change framework we utilized included six conditions, or areas, of systems change: policies, practices, resource flow, relationships and connections, power dynamics, and mental models (Kania et al., 2018). Change efforts often focus on the more explicit, structural areas of policies, practices, or resource flow, without addressing the implicit, underlying relationships, power dynamics, and mental models (Kania et al., 2018) that are critical to create sustained change. The Network’s approach of having participants engage in each of these six areas helped participants explore their own system more deeply.

The systems change framework was used in combination with a theory of change framework to visually explain how and why acting on identified leverage points would transform STEM teaching and learning at each institution (Rogers, 2014). Participants developed logic models that incorporated inputs, activities, outcomes, and impacts (Supplementary Material). Inputs are the financial, human, and material resources that, if we have them, allow us to undertake activities to address identified leverage points and contribute to desired outcomes. The outcomes ultimately contribute to the desired impact, in our case transforming undergraduate STEM education. Each box in the resulting logic model can be examined for what evidence is required to verify whether and how much change is occurring. Each arrow can be examined for leaps in logic, assumptions that are too big or include unwarranted risks. The logic models developed by each institution, while all having the same ultimate impact, differed based on the circumstances of their institution. Each one included both explicit and implicit conditions.

Finally, the (STEM)^2^ Network’s activities leverage the emergent outcomes model by embracing the themes, ideas, and practices that arise from the diverse group. As such, the Network engages the participants as agents of change. We focus on both the individual and the environments and structures in which those individuals exist since sustainable reforms necessitate change at both levels. As part of the Network, the use of emergent outcomes has allowed the Working Groups to develop their own goals and objectives, and the means of accomplishing them.

#### (STEM)^2^ Network groups

The (STEM)^2^ Network involves (1) multi-disciplinary and multi-institution Working Groups with concrete projects and (2) multi-disciplinary home institution groups. The goal was to subdivide the large Network into smaller Working Groups to allow the development of collaborations as participants rallied around projects related to the overarching Network goals. Participants self-selected which of three Working Groups were of interest to them.

##### Working groups

**Fig. 4:**
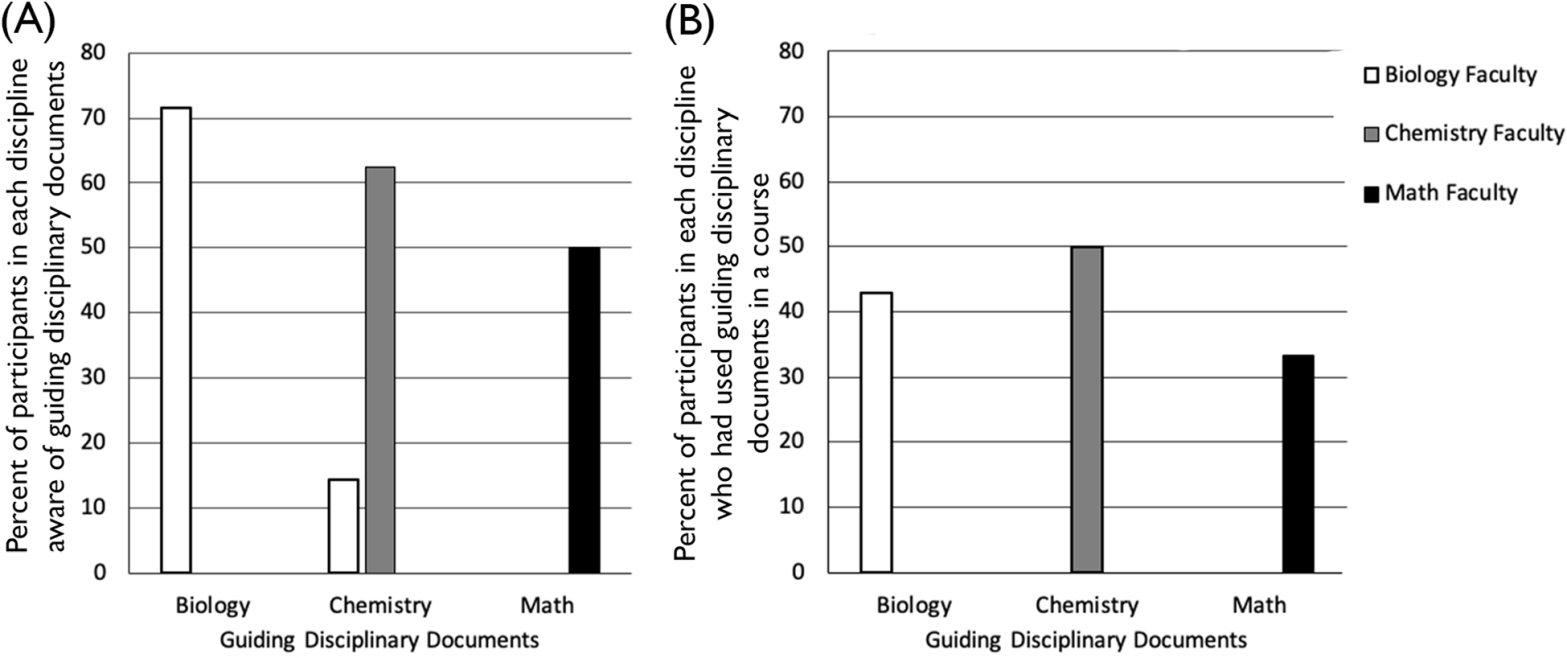
(STEM)^2^ Network Participants’ Awareness and Use of Guiding Documents. Percent of participants in biology (*N* = 7), chemistry (*N* = 8), and math (*N* = 6) who, prior to joining the (STEM)^2^ Network, were **a** aware of guiding documents in each discipline and **b** had used guiding documents in developing a course

The *Guiding Documents Working Group* aligned the disciplinary guiding documents that describe the concepts and competencies identified by disciplinary experts as critical for an undergraduate degree in each discipline (Table 1). While faculty may be familiar with the guiding documents of their own discipline, they are less likely to be familiar with those of others (Fig. 4). In this way, the interdisciplinary Guiding Documents Working Group addresses the effects of disciplinary silos by aligning the guiding documents to identify concepts and competencies that overlap between disciplines. Their work provides the foundation for on-going pedagogical collaboration across disciplines within the Network. One goal is to transform STEM curricula to shift student perception of their courses away from that of a series of disparate courses to a series of intentionally integrated, cross-linked, and cross-referenced curricula.

**Table 1:** Examples of guiding disciplinary documents in biology, chemistry, and math

Biology	Vision & Change (American Association for the Advancement of Science, 2011), BIO2010 (National Research Council, 2003), BioCore Guide (Brownell, Freeman, Wenderoth, & Crowe, 2014)
Chemistry	ACS Guidelines for Bachelor’s Degree Programs (American Chemical Society, Committee on Professional Training, 2015), ACS Assessment Tool for Chemistry in Two Year College Programs (American Chemical Society, Society Committee on Education, 2015)
Math	2015 CUPM Curriculum Guide to Majors in the Mathematical Sciences (Schumacher & Siegel, 2015), Key Mathematical Concepts in the Transition from Secondary School to University (Thomas et al., 2015)

The *Curriculum Alignment Working Group* focused more specifically on if and how courses aligned between our institutions. While formal articulation agreements exist between these institutions to facilitate awarding of transfer credit to students, communication at the faculty level regarding the course and program learning goals and changes in those goals through time was rare or nonexistent. Aligning learning goals across courses taken in the first 2 years at the 2- and 4-year schools provides a mechanism to re-assess and possibly re-align articulation agreements. The relationships built between faculty teaching these courses increases dialogue with the goal of better-supporting students who transfer from the 2- to the 4-year schools.

The *Systems Mapping Working Group* is developing a model that incorporates institutional data to identify barriers to student success that may not be readily evident. The model should be adaptable to any institution. Combining institutional systems maps (Supplementary Material) with the model will allow us to identify all barriers, both actual and perceived, and use those to identify key leverage points for change.

##### Institutional Groups

The goal of the Institutional Groups is to interrogate their institution’s structure, policies, and practices with the purpose of identifying and developing inclusive pathways to STEM student success. The Institutional Groups bridge disciplinary silos that exist at all of our institutions. The institutional systems mapping (Supplementary Material) that began at the full Network meeting sparked rich discussions about STEM education with colleagues across disciplines. The maps have created new avenues for communication that did not previously exist. This communication both revealed disconnects and promoted the sharing of initiatives occurring in our respective departments.

### Systemic challenges that the Network addresses

The (STEM)^2^ Network recognizes that sustained transformation necessitates collaboration across both disciplines and institutions. Therefore, the Network creates new connections and strengthens existing ones between faculty from different disciplines and institutions. The mental models underlying institutional structures resist change. Therefore, the Network engages faculty as stakeholders in the change process, equipping them with skills to create sustainable changes in institutional structures. In addition, transformation must occur from within the current paradigm. Therefore, the Network paradoxically equips faculty to change the system while simultaneously supporting them within the current system.

#### Disciplinary silos

An undergraduate degree in any particular STEM discipline requires classes across several disciplines. For example, biology majors take chemistry, physics, and math; chemistry majors take math and physics; and physics majors take math. In fact, the strategic revision of STEM courses taken *outside* the primary field of study positively impacts student learning in the *primary* field of study (Fisher, Fairweather, & Amey, 2001). Therefore, we should coordinate across the disciplines that students experience for a particular major.

The (STEM)^2^ Network engages faculty from across STEM disciplines to interrogate our curricula, classroom, and laboratory practices. Faculty are then able to identify opportunities to be intentionally interdisciplinary within our discipline-specific courses to show students that what they learn in one course is relevant in others. The multidisciplinary pedagogical collaborations created by the Network will have a greater positive impact on STEM student success relative to changes made within a single course or discipline.

#### Institutional silos

Institutional silos must be bridged for two reasons: first, an undergraduate degree often spans more than one institution with students transferring from 2- to 4-year schools, and second, institutional silos impede widespread transformation of the culture of higher education. With 5.7 million students enrolling in 2-year schools, and with that number projected to increase through time (Hussar et al., 2020), it is more important than ever to support their transition. Students who begin their college education at community colleges are less likely to attain a bachelor’s degree compared to students who begin their education at 4-year institutions (Wang, 2009). Some of the drivers of this trend are misalignment of curricula and gaps in advising that result from the lack of communication, both within an institution and between institutions (Wang, 2020). Therefore, communication across institutions is critical to help students successfully transfer, complete the 4-year degree, and enter a STEM career.

Although the community colleges in the Network are geographically close to the 4-year schools, and articulation agreements exist, the dialogue between faculty at the institutions regarding curricular pathways and courses is limited. The result is that the on-the-ground student experience of moving between the curricular pathways is fragmented and does not receive the attention it deserves. The (STEM)^2^ Network creates new avenues of communication across these institutions to improve the student transfer experience and likelihood of degree attainment.

Beyond considering collaboration between 2- and 4-year institutions, collaborations between similar institution types accelerate the transformation of undergraduate STEM education. Inter-institution collaborations leverage the knowledge, creativity, and experiences of the faculty to more fully address the higher education landscape as opposed to a single institution. In times of crises, such as the COVID-19 pandemic, having open, existing lines of communication across institutions is beneficial because it promotes the sharing of pedagogical and technological practices across a wider pool of instructors and helps those instructors benefit from mutual support.

#### Institutional transformation

The existing higher education system was not designed to handle the volume or diversity of people, in all aspects of the term, including but not limited to age, race, culture, and career goals, now seeking a college education (Trow, 2007). Through much of the nation’s history, higher education served primarily wealthy, white male students. Following World War II, college enrollments increased and community colleges became widespread, bringing access to higher education to more students and to students interested in entering a broader array of careers. More recently, the model for higher education is shifting towards a universal model as nearly all children are expected to attend college. With this brings a further expansion of the diversity of students, their needs, expectations, and goals. Higher education institutions are now expected to address issues of social mobility and equity of access for students. These factors combine to necessitate an institutional culture and structure that is fundamentally different from the original model. While institutions have shifted the original model through time to accommodate changes in the number and demographics of students enrolling, the fundamental culture of most institutions has not changed (Trow, 2007). This makes sustained transformation challenging.

The sustained transformation will require a systemic, rather than piecemeal, approach that addresses higher education’s core work processes of teaching, research, and service (Duffy & Reigeluth, 2008). It requires that faculty be empowered as change agents by developing their ability to assess institutions and by increasing their knowledge of institutional change processes. Furthermore, faculty must have the skills to work beyond their individual classrooms since *sustained* transformation requires work at the institution level to transform policies, practices, and culture. Since it is unlikely that any one individual could affect widespread institutional change, transformation efforts must be collaborative and participatory at all levels. The (STEM)^2^ Network, by engaging faculty stakeholders in collaborating to address classroom, disciplinary, institutional, and inter-institutional levels (Fig. 1), addresses the key components for sustained transformation identified by Duffy and Reigeluth (2008). The long-term goal is for faculty stakeholders to utilize the skills and products developed as they participate in the Network to engage with administrators including academic advisors. By working together, they will create strong, broadly reaching institutional changes.

#### Transformation from within the current paradigm

The current system’s bias towards rewarding research and publications as indicators of success, as opposed to teaching or service contributions (Anderson et al., 2011; Splitt, 2003), presents a particular challenge to the ability of faculty to address the transformation of undergraduate STEM education. To meet this challenge, the Network provides research, publication, and funding opportunities for Network participants. The Network bridges traditionally siloed disciplinary research foci by offering opportunities to collaborate on multidisciplinary education research projects. All Working Groups are encouraged to frame their work within the context of publishable products that reach broad audiences. For example, one Working Group is preparing a manuscript written by chemists and biologists from all five Network institutions; a manuscript that emerged as a result of their Network participation. More concretely, the Network supports publications by covering publication costs, which can be quite expensive. In the same vein, these collaborations lay the groundwork for the submission of grant proposals related to the transformation efforts driven by the Network. In this way, participants are rewarded within the existing system as they work to transform this system (Boyer et al., 2015).

A number of excellent programs exist to support faculty in transforming their individual classrooms (for example, Summer Institutes on Scientific Teaching (https://www.summerinstitutes.org), Mobile Summer Institutes on Scientific Teaching (https://www.summerinstitutes.org/mobile-institutes), the POGIL Project (pogil.org), and SENCER (sencer.net)). The PULSE Network (pulse-community.org) has a broader focus of transforming biology curricula, while PKAL (https://www.aacu.org/pkal), HERS (https://www.hersnetwork.org/), ASCN (ascnhighered.org), and SEAChange (https://seachange.aaas.org/) focus on change at the program and institutional levels. The (STEM)^2^ Network leverages the contributions of these programs, as many of our participants are their alumni. The Network extends their impact by integrating work across levels (Fig. 1) to ensure coordinated and sustained transformation.

## Methods

Two of the goals of the Network are to empower participants to become change agents and encourage members to collaborate across disciplines and institutions. Because the Network has a limited number of initial participants, we looked to see how individual participants reported changing through time. This research was approved by the Institutional Review Board (reference number: IRB-FY2020-612), and informed consent was provided by all study participants.

**Fig. 5:**
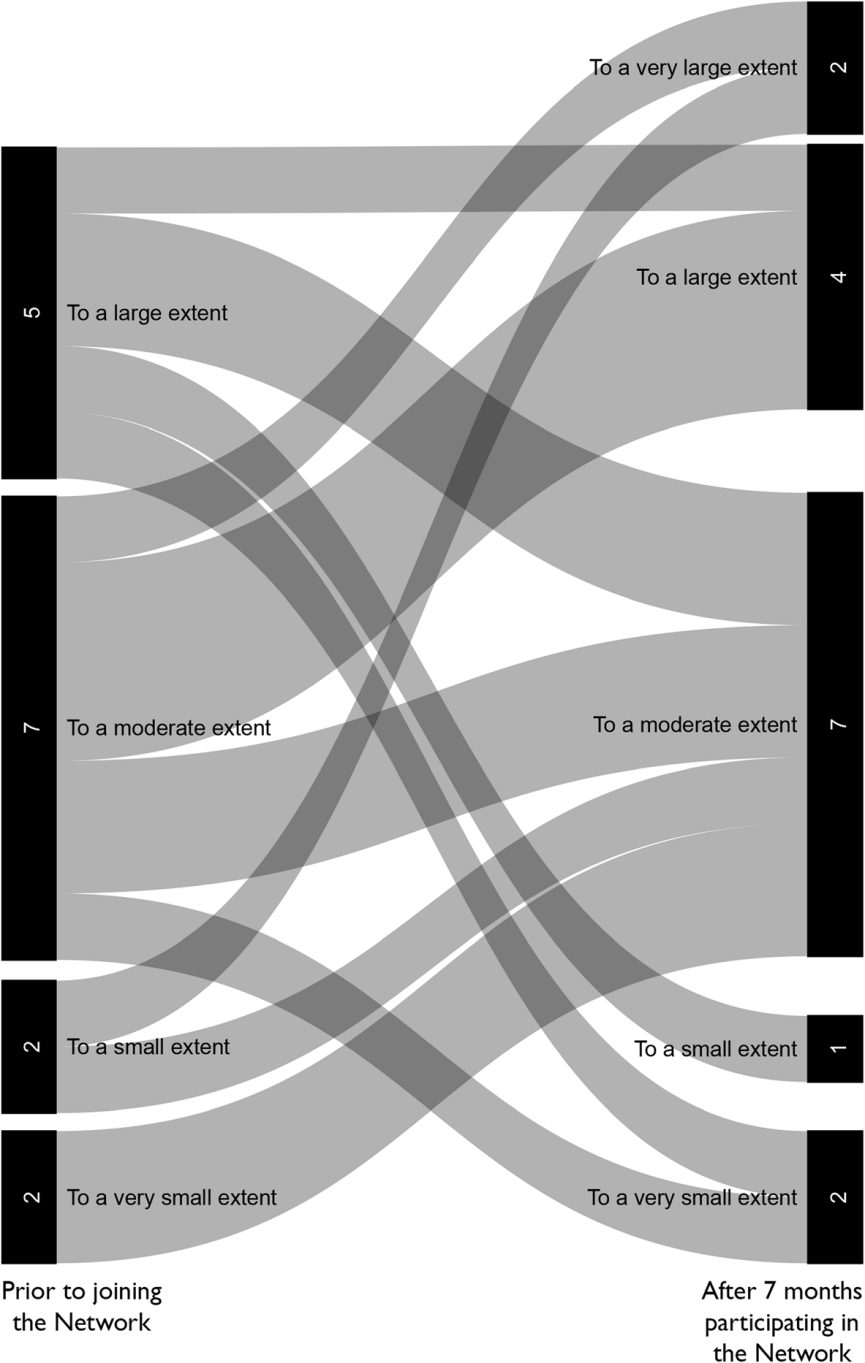
Change in (STEM)^2^ Network Participants’ Feelings about being Change Agents. Change in reported sense of how prepared participants felt to be a change agent for STEM education reform over 7 months in the (STEM)^2^ Network. Left most bars are prior to joining the Network. Right most bars are after 7 months in the Network

**Fig. 6:**
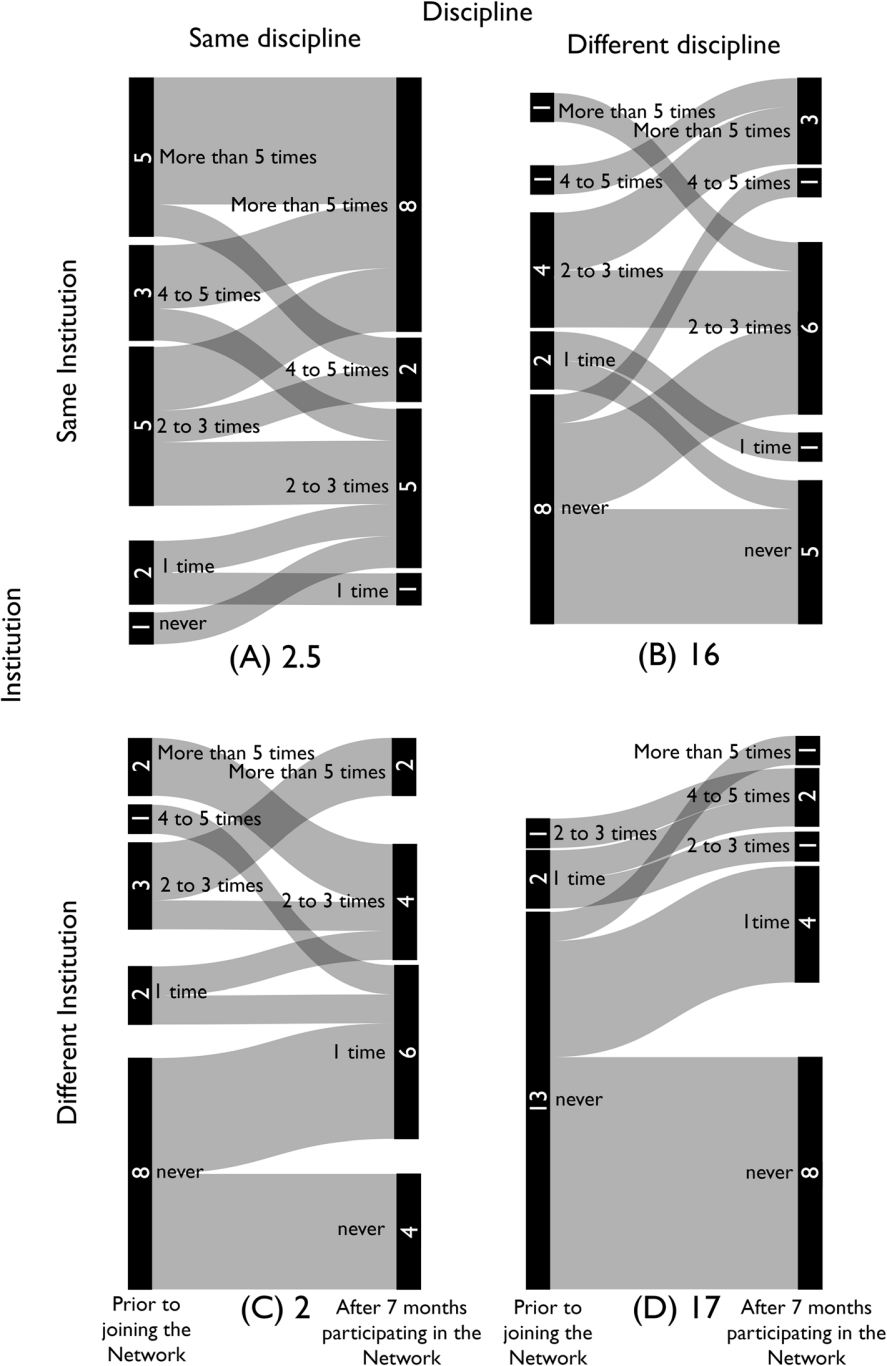
Increase in Collaborations with Colleagues. The number of times participants reported collaborating with colleagues prior to joining the Network (left bars in each Sankey diagram) and after 7 months of participating in the (STEM)^2^ Network (right bars in each Sankey diagram) in **a** the same discipline at their home institution, **b** different disciplines at their home institution, **c** the same discipline at other institutions, and **d** different disciplines at other institutions. The numbers below the figures represent the increase in participants’ interactions from prior to joining the Network to 7 months after joining the Network. Interactions were calculated by multiplying the number of participants in each collaboration category at each time point by 0 (for never), 1 (for 1 time), 2.5 (for 2–3 times), 4.5 (for 4–5 times), and 6 (for more than 5 times)

Participants were surveyed prior to the first Network meeting and after 7 months in the Network. They were asked (1) how prepared they felt to be a change agent for STEM education reform; (2) how many times in the past year they had collaborated with colleagues (a) in their discipline at their institution, (b) in other disciplines at their institution, (c) in their discipline at other institutions, and (d) in other disciplines at other institutions; and (3) if they were aware of or used guiding disciplinary documents in biology, chemistry, and mathematics in developing or teaching courses. We mapped the resulting data for (1) and (2) using Sankey diagrams (Figs. 5 and 6). With respect to collaborations, we calculated the total number of interactions by multiplying the number of participants in each collaboration category at each time point by 0 (for never), 1 (for 1 time), 2.5 (for 2–3 times), 4.5 (for 4–5 times), and 6 (for more than 5 times). We then compared the initial number of collaborations with the number of collaborations reported after 7 months’ participation in the Network.

Participants also responded to open-ended prompts 10 months after joining the Network. We used these responses to explore participants’ motivations underlying their decision to join the Network, why they continue to participate in the Network, and how the Network contributes to their professional growth.

## Results and discussion

Participation in the (STEM)^2^ Network increased feelings of preparation to be change agents for STEM education reform. Of the 16 participants completing both a pre-Network and a mid-Network survey, the number of participants who felt they were prepared to be a change agent to a small or very small extent decreased while the number who felt they were prepared to be a change agent to a large or very large extent increased (Fig. 5). This increase in the sense of empowerment is a result of the Network’s intentionality with respect to faculty development in this area. This is an accomplishment after only 7 months of Network involvement that were interrupted by the COVID-19 pandemic.

Participation in the Network increased collaboration within and across both disciplines and institutions. The number of interactions within and across disciplines and institutions increased since the beginning of the Network (Fig. 6). More importantly, the number of participants who had not collaborated with colleagues in the past year decreased. More participants now report collaborating at least once, especially with colleagues in different disciplines (Fig. 6). This is a result of all Network meetings, whether full Network, Working Groups, or Institutional Groups being intentionally interdisciplinary and, for full Network and Working Groups, intentionally inter-institutional.

The Network promotes interdisciplinary collaboration. The greatest increase in collaboration occurred across disciplines both within (Fig. 6b) and across (Fig. 6d) institutions. Two goals of the Network are to create enduring pedagogical collaborations (1) across STEM disciplines and (2) between community colleges and 4-year institutions. Barriers to achieving these goals are the culture of siloed disciplines and institutions and a lack of time to engage in these collaborations due to the focus on scholarship at 4-year institutions and heavy teaching loads at 2-year institutions (Anderson et al., 2011; Brownell et al., 2014; Splitt, 2003). The Network bridges these silos, providing the time and framework to engage with colleagues.

The Network lays the groundwork for pedagogical collaboration across disciplines. Participation in the Network increased the number of participants who were aware of both their own and other disciplines’ guiding documents (Table 1). Prior to joining the Network, most participants were only aware of the documents related to their own discipline with few aware of documents from other disciplines (Fig. 4a). Participants who had used the documents to construct a course only used documents from their own discipline, never those from other disciplines (Fig. 4b). Purposefully introducing the documents to participants at Network meetings ensured that all participants are now aware of and have interacted with guiding documents from all three disciplines. The on-going work of the Guiding Documents Working Group to align the concepts and competencies across the disciplines will facilitate participants being explicitly interdisciplinary when teaching courses. We will follow-up on this work by tracking the extent to which participants utilize these documents and their alignment of them in their teaching.

Four primary motivations drove participants to join the Network. Participants consistently stated that they joined to improve STEM education, to improve the transfer student experience, to collaborate with colleagues across disciplines and institutions, and because they respected the leadership team (Table 2). These motivations align with the goals of the Network. This highlights the importance of clearly stating the goals of any particular network to ensure that participants have reasonable expectations of the outcomes and benefits of participation. Responses further demonstrate the importance of relationships and people. In this case, participants were motivated to join due to the connections with and reputation of Network leaders.

**Table 2:** Participant responses to survey questions regarding motivations to join the (STEM)^2^ Network, and sustained participation in and professional growth resulting from participation in the Network

Survey question	Selected participant responses
What motivated you to join the Network?	Interest in advancing teaching in higher education; respect for the PIs’ ideas and abilities
	A desire to improve success and experiences for the many students who transfer from 2 year schools to our 4 year school (and have a very tough transition!)
	I chose to join the (STEM)^2^ network out of a desire to improve inclusivity within STEM through data-driven and collaborative methods. Our University is a small sample-size in a greater pool of local institutions that all serve similar populations of students. It makes sense to combine our experiences to identify areas where we can allocate resources to benefit our STEM majors the most.
What sustains continued participation in the Network?	Working with amazing people - from co-PIs to members of my working group - these individuals are among the most cooperative, most selfless people I have had the pleasure of knowing. Especially during some of the dark days of COVID, these individuals were always positive, always sharing. I felt energized after our meetings.
	Having the chance to collaborate
	The network seems to be working.
	I feel that this network has broadened my view of how STEM majors are designed. I look forward to seeing our project continued, as I think this will benefit our students and our ability as instructors to advocate for them.
	Professional development and potential publications
	Excellent continuing interaction with peers at neighboring institutions working toward helping STEM students in a more coordinated fashion
How does the Network contribute to your professional growth?	Get different perspectives from faculty at other institutions; think about things I haven't thought about before (e.g., network mapping); see approaches of PIs and others to scholarship in a different field from basic science
	I've really enjoyed interacting with my peers at other institutions (and even at my own institution across departments). Those interactions and conversations, on their own, are extremely valuable. I'm confident, that with time, we will be able to propose and implement some thoughtful curriculum changes that will hopefully help our students.
	Yes, even during this very challenging 2020 year, the Network helped me evolve as a teacher and mentor of undergraduate STEM students. Also made me feel part of a bigger movement than that of my classroom or my department.
	Spurs thinking of new ways to look at old, systemic problems.
	My skill set in modeling has definitely expanded, and I feel like I have collaborative contacts at multiple institutions in my local area. The power of this network to bring together faculty and administrators from across STEM disciplines is really important, and it gives me a chance to form new research collaborations with other faculty nearby.

Participants continue to engage in the Network because of the collaborations created, opportunities for professional growth, opportunities to improve STEM education, and a sense that the Network is functioning as intended (Table 2). Almost every participant mentioned collaborations specifically, for example, “having the chance to collaborate,” or indirectly, for example, stating that their perspectives had shifted due to interactions with other colleagues. This again emphasizes the importance of relationships and people. In this case, the positive interactions among the participants sustain their continued involvement in the Network. Participants also remain in the Network because they gain practical skills, such as systems mapping, and tangible results, such as publications. They perceive that the collaborations support their ability to improve STEM education. As with the motivations to join the Network, constantly and clearly articulating the goals of the Network and aligning Network activities with the goals is critical to sustained participant involvement.

The Network contributes to participant professional growth. Participants report that the Network supports their evolution as educators through interdisciplinary and inter-institutional collaborations. The Network has also shifted their perspectives on STEM education across disciplines and institution types, and offers new skills, such as systems mapping, that contribute to their ability to be agents for STEM education reform. These elements of professional growth directly align with the Network’s goals of promoting collaboration and empowering faculty to transform undergraduate STEM education.

Overall, the (STEM)^2^ Network is progressing towards achieving its goal of transforming undergraduate STEM education. It is reaching milestones of empowering faculty to create change beyond their individual classrooms, promoting new collaborations among biology, chemistry, and math faculty at 2- and 4-year institutions, and creating enduring pedagogical collaborations across STEM disciplines.

### Lessons learned

Several lessons have emerged since forming the (STEM)^2^ Network that could be applicable for others. First, existing relationships across the institutions, even the weak ones, acted as catalysts to form more and stronger connections. Second, the overlap in the concerns and constraints due to sharing demographically similar students and working in the same geographic region provided multiple opportunities for discussion and collaboration. This became particularly evident during the COVID-19 pandemic as Network participants, having already formed stronger relationships, were able to communicate and share relevant ideas and resources. Third, we discovered that participants want to meet regularly and that it is easier to find a full day for Network meetings during January intersession and summer than for participants to schedule even one hour during the semester for a Working Group meeting. Finally, iterative feedback and making adjustments are critical to the success of the Network, especially in its nascent stages.

Looking beyond our immediate set of institutions, we envision that this Network model is adaptable to accommodate other institution types. For example, regional comprehensive universities, institutions in more rural areas, and institutions serving different student populations may have different strengths and challenges relative to the institutions currently in our Network. All of these institution types would likely benefit from enhancing relationships across disciplines and with other institutions. The (STEM)^2^ Network provides a model to achieve these results.

The nature of the theoretical frameworks underlying the (STEM)^2^ Network and the structure of inter-disciplinary and inter-institutional groups is flexible and adaptable. Indeed, the systems mapping component allows any institution or set of institutions to identify their specific strengths and areas for growth including areas of overlap between institutions. Systems mapping allows institutions to make informed decisions about the structure and direction of their work to suit their unique set of needs. The emergent outcomes approach allows any network to tailor the focus of their efforts to the ideas that arise from their participants. Utilizing the principles of a Community of Transformation, any network can simultaneously address both individual faculty development and promote transformations in the broader system. Structuring a network with intentionally interdisciplinary and inter-institutional working groups brings diverse faculty together around a common goal, bridges disciplinary silos at one institution, and bridges silos across institutions.

## Conclusion

If the goal is to transform undergraduate STEM education to increase the number and diversity of people entering STEM careers, we must fundamentally transform the STEM higher education system. This transformation must embrace the pathways model to a STEM career, which is more realistic and inclusive than the more traditional pipeline model (Cannady et al., 2014). To achieve this goal, we must equip faculty, the frontline stakeholders who interact with students every day, to transform the system from the inside. We must build relationships and collaborations across disciplines and institutions to effect widespread transformation. The systemic transformation the Network hopes to achieve is challenging because it pushes against the existing culture in higher education. The (STEM)^2^ Network provides a model construct to meet this challenge via its foundation on a Community of Transformation model, use of emergent outcomes to guide Network activities, and utilization of systems design for organizational change to transform the complex higher education landscape.

## Supplementary Information

**Additional file 1.**

## Data Availability

The datasets used and/or analyzed during the current study are available from the corresponding author on reasonable request.

## Abbreviations

ACS: American Chemical Society
ASCN: Accelerating Systemic Change Network
CoT: Communities of Transformation
HERS: Higher Education Resource Services
NCC: Nassau Community College
NSF: National Science Foundation
PKAL: Project Kaleidoscope
POGIL: Process-Oriented Guided Inquiry Learning
PULSE Network: Partnership for Undergraduate Life Sciences Education
QCC: Queensborough Community College
RCN-UBE: Research Coordination Network - Undergraduate Biology Education
SEAChange: STEMM (Science Technology Engineering Math and Medicine) Equity and Achievement Change
SENCER: Science Education for New Civic Engagements and Responsibilities
STEM: Science, Technology, Engineering, and Math
(STEM)^2^: Sustainable, Transformative Engagement across a Multi-Institution/Multidisciplinary STEM
