# Supplementary material for: The (STEM)2 Network: a multi-institution, multidisciplinary approach to transforming undergraduate STEM education
Source: Int J STEM Educ. 2021 Jan 29;8(1):3. doi: 10.1186/s40594-020-00262-z (PMC7843166; doi:10.1186/s40594-020-00262-z)
Supplement: Supplementary file 1 — Additional file 1. [file 40594_2020_262_MOESM1_ESM.pdf]

Supplementary Material

Institution 1: Rich Picture

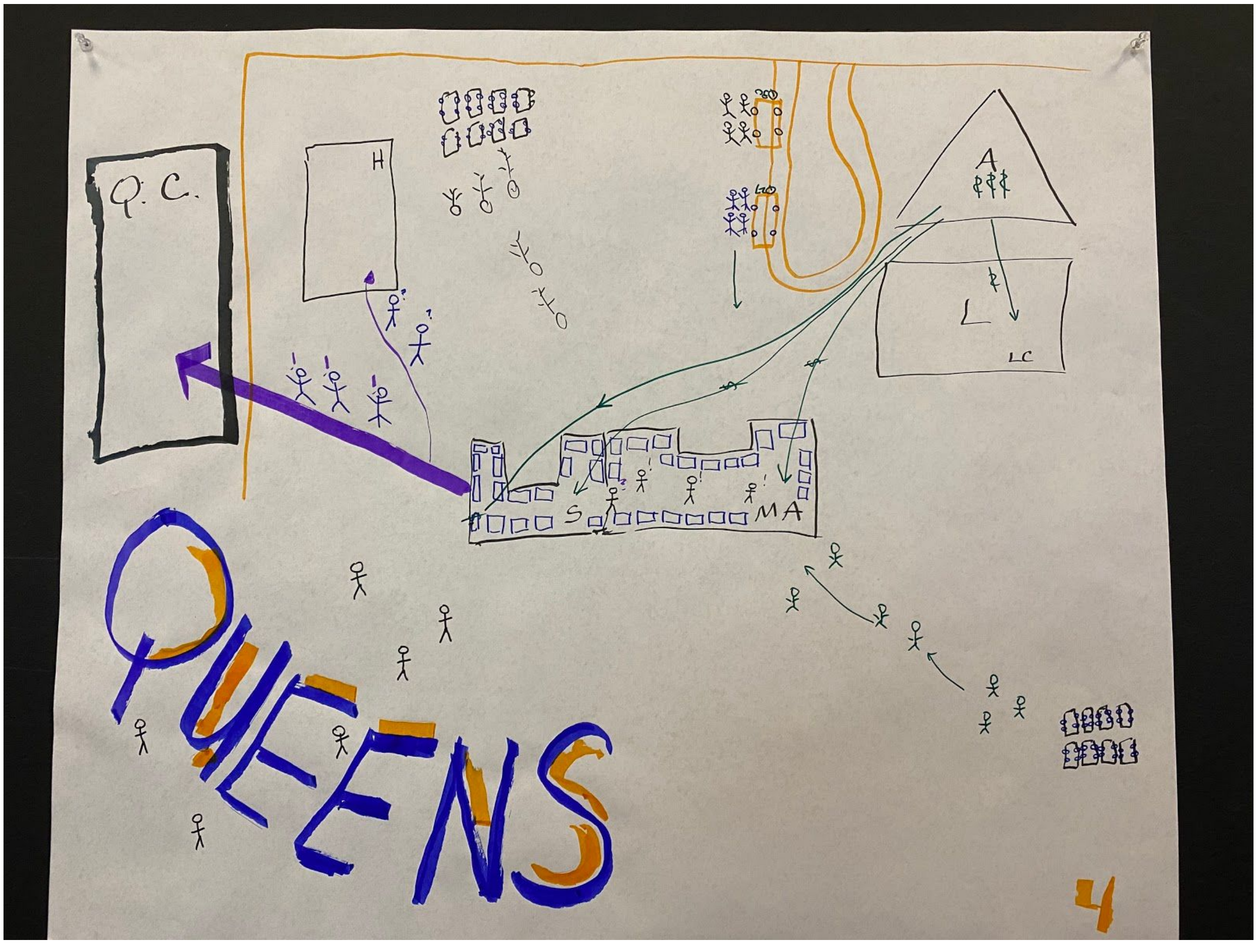

Institution 1's rich picture diagrams the route students take to obtain their degrees.

Institution 1: Influence Diagram

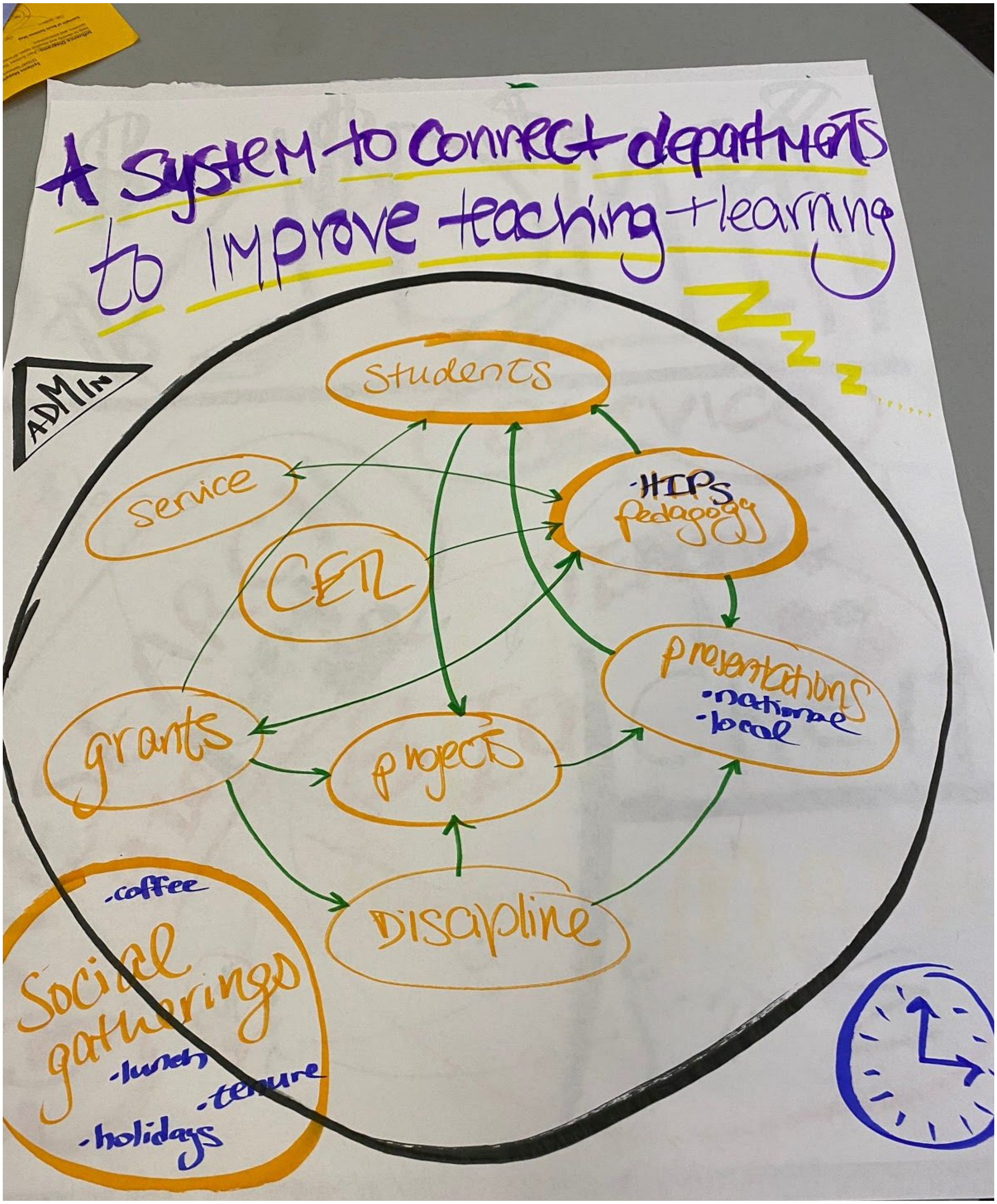

The influence diagram from Institution 1 identifies the connections and leverage points within their complex system.

Institution 1: Logic Model

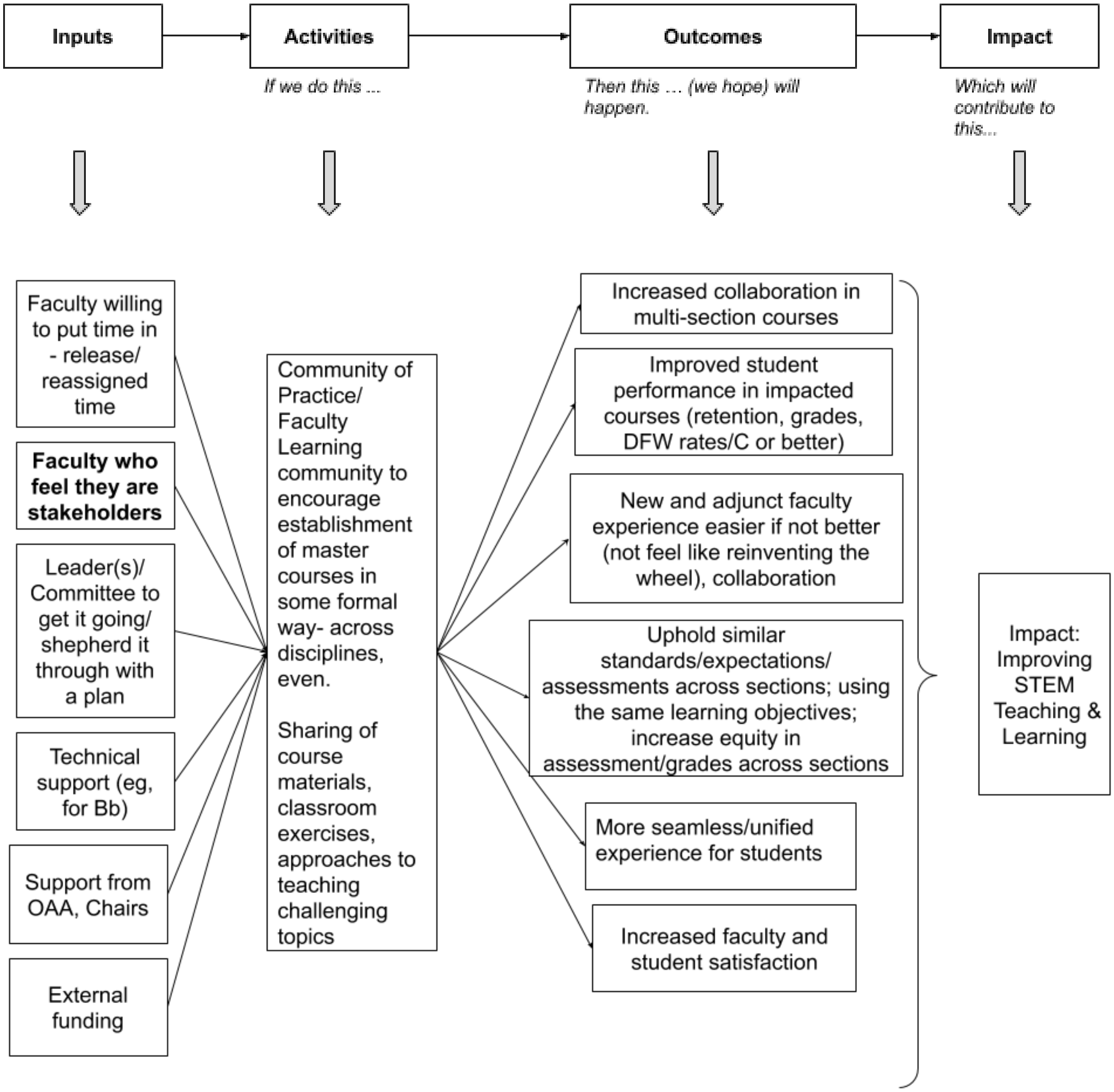

Institution 1's logic model starts with the activities of forming a Community of Practice/Faculty Learning Community. The Learning Community requires the inputs of the faculty and support. They anticipate improved student outcomes and satisfaction with the ultimate impact of improving STEM teaching and learning.

Institution 2: Rich Picture

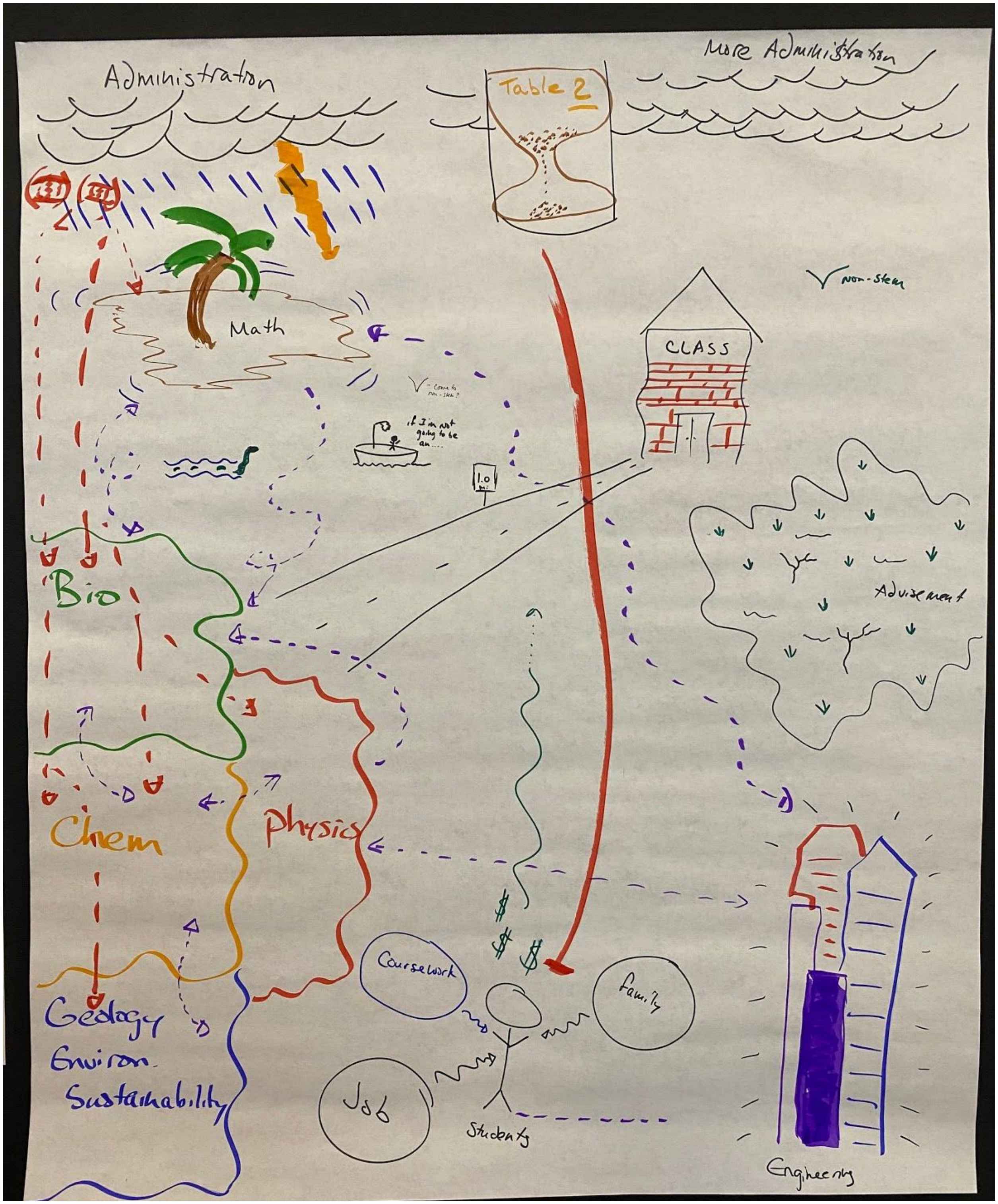

Institution 2's rich picture depicts the elements of their institution which influence STEM degree attainment.

Institution 2 Influence Diagram

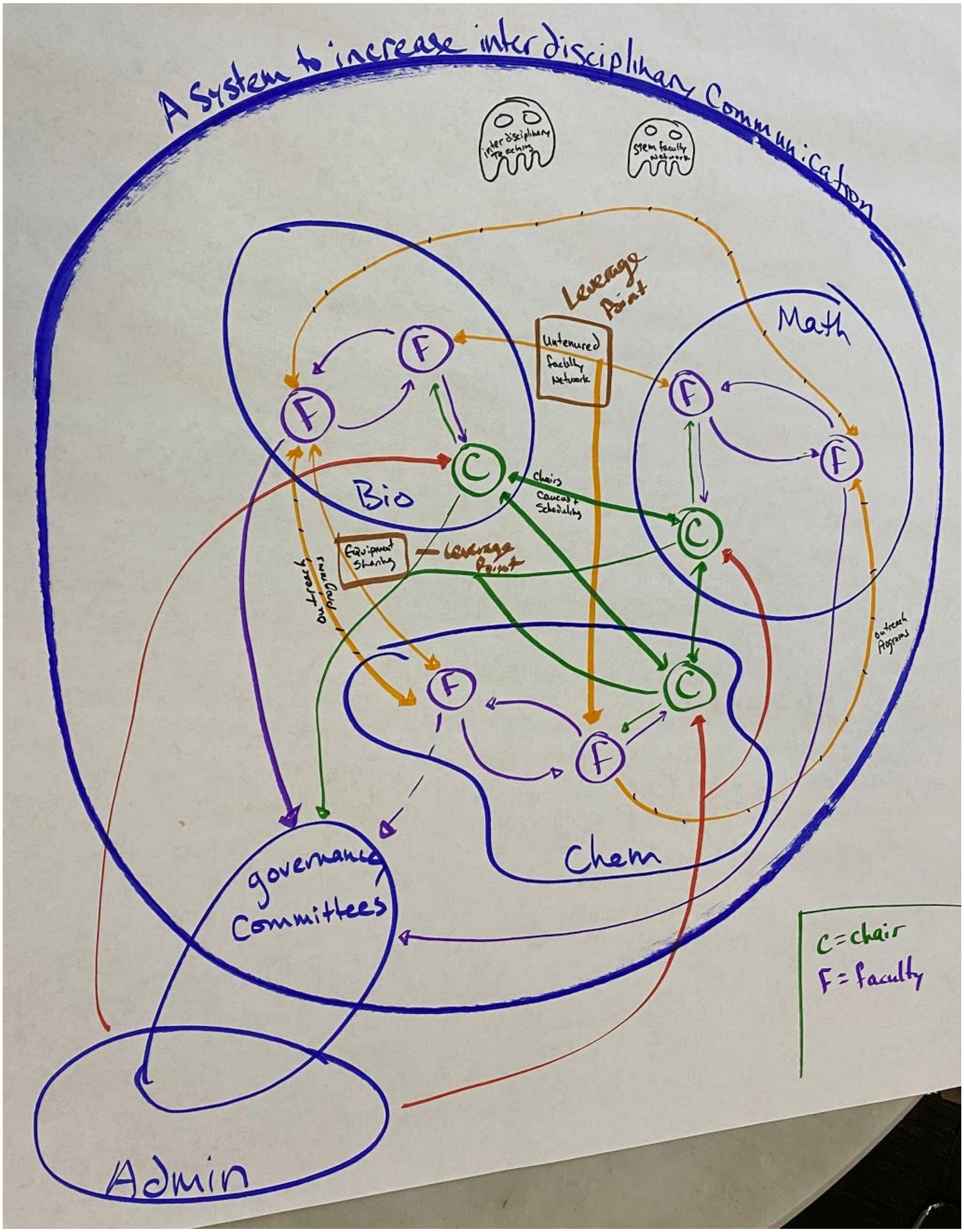

The influence diagram visualizes the connections between elements at their institution related to communication across disciplines required for a STEM degree.

Institution 2: Logic Model

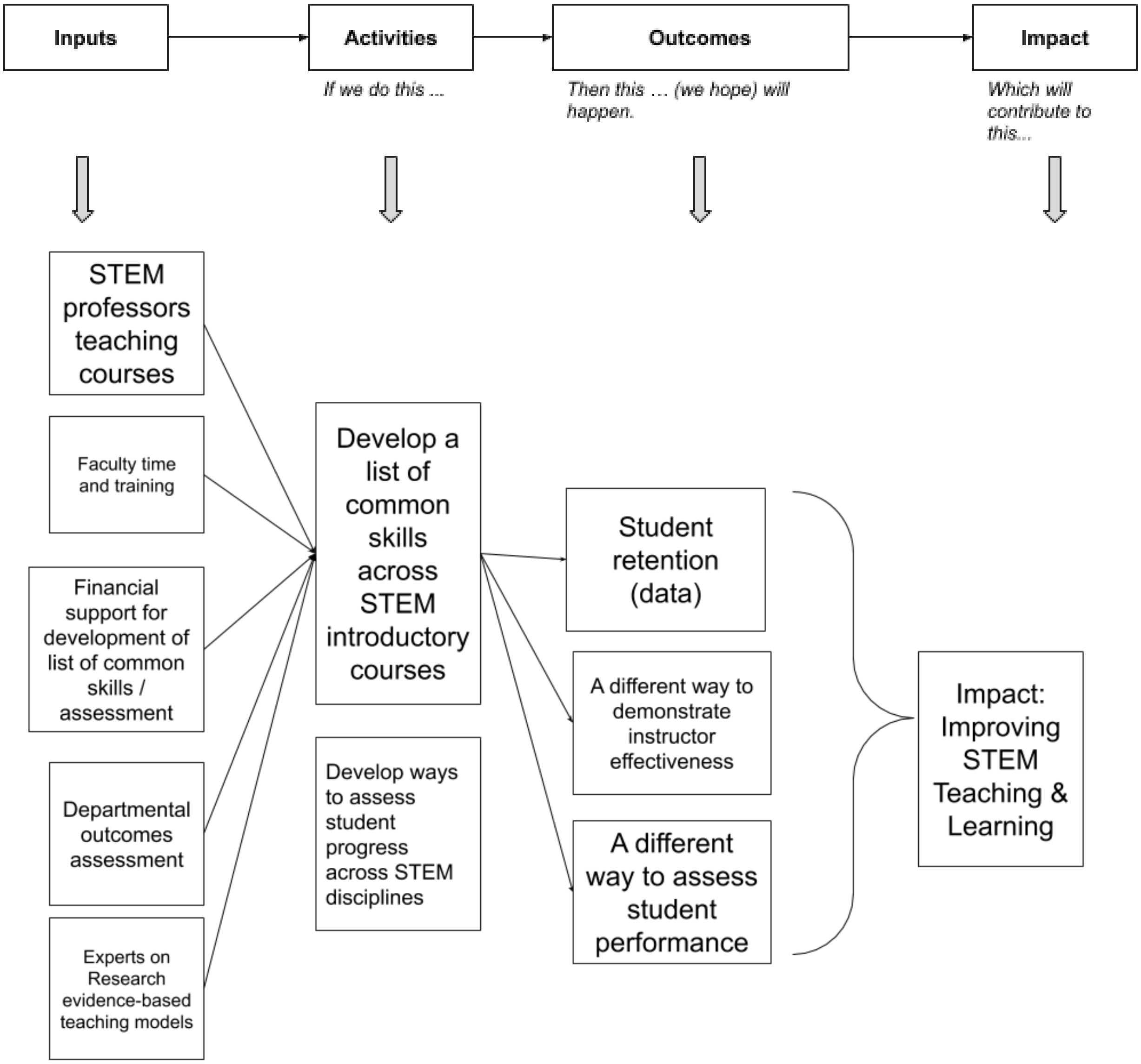

Institution 2's logic model starts with developing a list of common skills across STEM introductory courses. This activity requires multiple inputs from faculty, departments, and education research experts. They anticipate increases in student retention and alterations to the current system of evaluating instructor effectiveness as well as student performance. Ultimately, these will impact STEM teaching and learning at their institution.
